# An online survey of personal mosquito-repellent strategies

**DOI:** 10.7717/peerj.5151

**Published:** 2018-07-03

**Authors:** Emily Lucille Moore, Mary Alice Scott, Stacy Deadra Rodriguez, Soumi Mitra, Julia Vulcan, Joel Javierla Cordova, Hae-Na Chung, Debora Linhares Lino de Souza, Kristina Kay Gonzales, Immo Alex Hansen

**Affiliations:** 1 Department of Biology, New Mexico State University, Las Cruces, NM, United States of America; 2 Department of Anthropology, New Mexico State University, Las Cruces, NM, United States of America; 3 Departmento de Biolgia Geral, Universidade federal de Viçosa, Vicosa, Brazil; 4 Institute for Applied Biosciences, New Mexico State University, Las Cruces, NM, United States of America

**Keywords:** Survey, Mosquito, Repellents

## Abstract

**Background:**

Mosquito repellents can be an effective method for personal protection against mosquito bites that are a nuisance and carry the risk of transmission of mosquito-borne pathogens like *plasmodia*, dengue virus, chikungunya virus, and Zika virus. A multitude of commercially available products are currently on the market, some of them highly effective while others have low or no efficacy. Many home remedies of unknown efficacy are also widely used.

**Methods:**

We conducted a survey study to determine what kind of mosquito repellents and other mosquito control strategies people use. Our online survey was focused on unconventional methods and was answered by 5,209 participants.

**Results:**

The majority of participants resided in the United States, were female (67%), had higher education (81% had a university degree), and were 18 to 37 years old (50%). The most commonly used repellent was DEET spray (48%), followed closely by citronella candles (43%) and ‘natural’ repellent sprays (36%). We collected a plethora of home remedies and other strategies people use that warrant further research into their effectiveness.

**Discussion:**

Our study lays the foundation for future research in alternative, unconventional methods to repel mosquitoes that may be culturally acceptable and accessible for people.

## Background

Mosquitoes are vectors for infectious diseases that cause widespread epidemics and human morbidity (Brower, 2001). The pathogens transmitted by mosquitoes are quite diverse and include protozoans, arboviruses, and filarial nematodes (Fredericks & Fernandez-Sesma, 2014; Marquardt, 2004). These pathogens are taken up from one host and transmitted to another during the process of blood feeding. Mosquito-transmitted diseases put hundreds of millions of people at risk and still kill more than half a million people every year despite immense international efforts to combat them (Newby et al., 2016). Developing countries in tropical and subtropical regions bear the greatest burden with the majority of fatalities being young children (Snow et al., 2005).

The host-seeking behavior of mosquitoes as well as many other hematophagous arthropods depends heavily on their sense of smell. A battery of specific odorant receptors expressed in odorant receptor neurons within the antennae of mosquitoes enables them to detect a variety of chemical clues that are emitted by the host (Bohbot et al., 2007; Carey et al., 2010). Many of these attractants, for example CO_2_, organic acids, and aldehydes, have been identified and linked to specific receptor proteins (Beever, 2006; Chen & Luetje, 2014; Dong et al., 2013; Gopal & Kannabiran, 2013; Jones et al., 2012; Kumar et al., 2013; Nichols, Chen & Luetje, 2011; Suh, Bohbot & Zwiebel, 2014; Taylor et al., 2012; Turner et al., 2014; Xu et al., 2014). Olfactory receptor agonists as well as antagonists can impede the mosquito’s sense of smell and interrupt host-seeking behavior. Chemicals that elicit such responses are termed mosquito repellents.

Reducing the number of host-vector interactions is an effective way to reduce the spread of vector-borne diseases. Currently, only a small number of active ingredients in a large number of different commercially available formulations are widely used to protect humans from mosquitoes and other blood-sucking arthropods. DEET (N,N-Diethyl-meta-toluamide) is commonly used as an active ingredient in insect repellents (Hansen et al., 2014; Xu et al., 2014). Unsubstantiated fears of possible side effects of DEET have created a large market for “natural” DEET-free repellents with a variety of active ingredients. Picaridin, IR 3535, and a large assortment of essential oils, such as eucalyptus and lemongrass, are sold as sprays, creams, and integrated in wearable devices for repelling mosquitos (Xue, 2015). For a review of the long history and present use of plant extracts as commercial insecticides please see a recent review by Pavela (2016). A current text search on http://www.amazon.com with the search string “insect repellent” resulted in 22,950 hits.

Access to effective repellent products is often limited in developing countries. People with a high risk of vector-borne disease infections often have no or only insufficient means to protect themselves (Benelli & Mehlhorn, 2016). Across cultures, people use a variety of home remedies and traditional practices to repel mosquitos (Scandurra et al., 2014). Some of these remedies are likely highly effective, while others (Acheson, Plowright & Kerr, 2015; Clark et al., 2016; Scandurra et al., 2014) like DEET are expensive and scarce. In addition, some known effective methods, such as the use of mosquito nets, are impractical in particular situations and are therefore not used regularly by community members (Acheson, Plowright & Kerr, 2015; Clark et al., 2016; Scandurra et al., 2014). Several ethnobotanical research projects have focused on the traditional use of native plants as insect repellents (Pavela & Benelli, 2016; Tisgratog et al., 2016).

In a recent study, we have determined the efficacy of several commercial products, two fragrances, and a vitamin B patch in repelling mosquitoes (Rodriguez et al., 2015a ). The products were tested using a Y-tube olfactometer setup with *Aedes aegypti* (Linnaeus) and *Aedes albopictus* (Skuse), both major human disease vectors. Repellents with DEET or p-menthane-3,8-diol (PMD) as active ingredients had a prominent repellency effect over longer times and on both species. Some of the DEET-free products containing citronella or geraniol did not have any significant repellency effect. Interestingly, the perfume we tested had a significant repellency effect early after application. These findings were widely reported in the media and we received information from the public about other personal hygiene products that are used as mosquito repellents (see below).

In a follow up study, we performed attraction-inhibition assays using a taxis cage in a wind tunnel setting (Rodriguez et al., 2017). One person was placed upwind of the taxis cage and the mosquito movement towards or away from the person was recorded. The person was treated with various spray-on repellents or equipped with different ‘mosquito repellent devices’. We found that the spray-on repellents containing 98% DEET or 30% PMD had the highest efficacy in repelling mosquitoes compared to repellents with other ingredients. From the five wearable devices that we tested, only the one that releases Metofluthrin significantly reduced the numbers of attracted mosquitoes. A citronella candle had no effect. We concluded that many of the products that we tested that were marketed as repellents do not reduce mosquito attraction to humans.

The current study used an online survey to find unconventional methods for mosquito control and repellent practices that will be tested in future experiments. Here we define an unconventional method as one involving a product that is not commercially available worldwide, or involving products that are not used for their original purpose (i.e., dryer sheets). We also considered behaviors that are not obviously connected to mosquito control (i.e., drinking alcohol or eating bananas) as ‘unusual’. Links to the survey were distributed globally in an effort to capture a broad diversity of practices; however, outreach proved to be very complicated. The supplementary material shows the demographics of our study were biased; however, we did attain very useful qualitative input from our respondents.

## Methods

### Online survey

We developed a nine question online survey (see Supplemental Information 1). Demographic variables included gender, age, education levels and residence. Survey questions assessed people’s awareness of mosquitoes, general knowledge about mosquito-borne diseases and mosquito repellents and what mosquito control strategies people most frequently use. Two survey questions were specifically designed to gather unconventional mosquito repellent methods and strategies. The survey was available in English, Spanish, and Portuguese and was distributed in countries in which residents were likely to speak one of the above three languages. Convenience and snowball sampling were used.

### Data collection

The data was collected anonymously through the online portal Survey Monkey (Palo Alto, CA, USA). Participants were recruited through various electronic means including postings on Facebook or other social media, and email list serves. The survey was shared on Facebook groups most of which were academics-led with ‘entomology’ or ‘health’ in the title, with the assumption that these groups are interested in this topic resulting in more participation and shares. The survey was also provided to various universities through personal contacts of the authors. The authors also forwarded the survey to their personal email contact lists. Those who agreed to participate had the option to enter a drawing for one of 10 gift cards valued between $50 and $100.

### Data analysis

The data was exported into excel from Survey Monkey and categorized by manual inspection. For questions for which answers were provided, the Excel sort function was used to group and count identical answers. Answers to the open questions were analyzed by the authors and placed into different categories that we defined. Geographic region was determined by the continental location of the respondents’ current residence. Open-ended responses to the survey question regarding unconventional practices were categorized by type of mosquito control or repellent method.

### Ethics statement

The study was approved by the NMSU Institutional Review Board (IRB # 15378, approved July 10th 2017). Consent was obtained by participants’ selecting to continue on to the survey after reading a description of the study, including risks and benefits.

## Results

### Demographics

**Figure 1:**
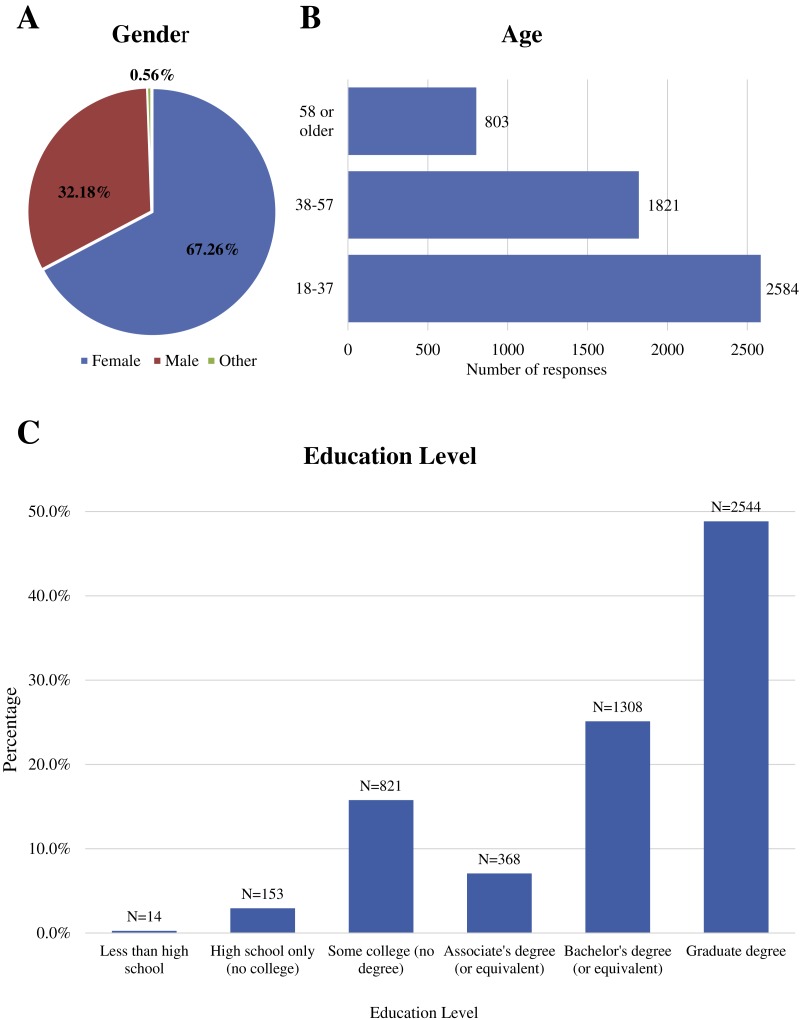
Demographics of study participants. (A) Gender of participants. (B) Age of participants in years. (C) Educational level of study participants.

We received a total of 5,209 responses to our survey (for raw data, please see Supplemental Information 2). Figure 1 shows the demographics of the survey participants, where 67.71% of participants identified as female, 31.7% as male, and 0.59% as other; the majority of respondents were 18 to 37 years , followed by ages 38 to 57 years with ages over 58 years being the lowest (*N* = 789); and almost 50% held a graduate degree. Listed in Supplemental Information 3 are the regions and countries in which the respondents stated they were currently residing at the time of the survey. Over 76% were from North America, with 70% of the total participants listing the United States. Europe had the next highest number of responses, totaling to a little over 7% of total respondents followed by Australia (6.3%), Asia (4%), Africa (3.8%) and South America (3.3%).

### Mosquito repellent methods

**Figure 2:**
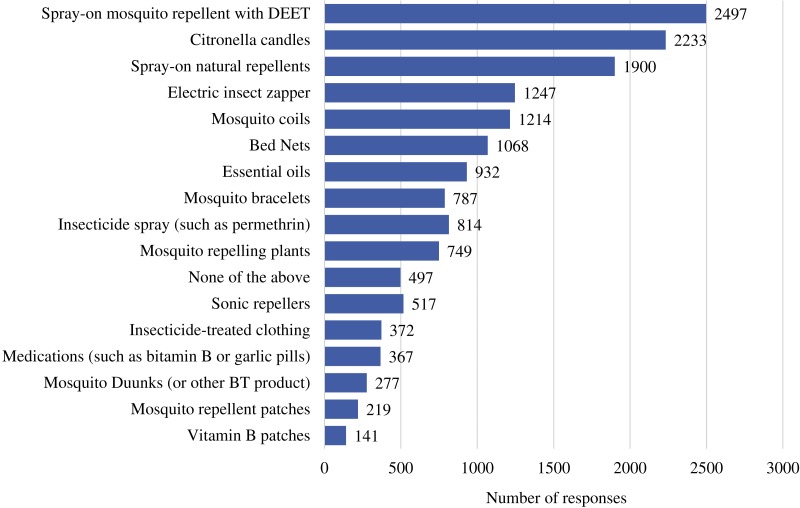
Commonly used mosquito repellent methods. The number of each response from one of the multiple choice questions.

We chose 13 mosquito repellent/control methods and asked respondents to select all methods they had ever used. Answer choices can be seen in Fig. 2. Out of the 5,209 total respondents, 4,773 responded to the multiple choice question. The most common repellent method chosen by the respondents was spray-on mosquito repellent with DEET, closely followed by citronella candles and ‘natural’ spray-on repellents (Fig. 2). In the open ended question picaridin was mentioned by 59 participants and IR3535 only once.

**Table 1:** Table showing mosquito repellent methods & strategies. Shown are mosquito repellent methods that were collected through the open ended survey question, along with the way of administration or preparation and the region(s) they were listed from. The methods/strategies are listed according to the numbers of references (from high to low).

Responses	Administration/preparation	Region	Ref. No.
Telephone app (sound)	Application	Sweden	
Cut down on sugar	Avoidance, Ingestion	USA, Denmark	
No meat consumption	Avoidance	UK	
Bananas	Avoidance, Ingestion, Topical, Unspecified	USA, Canada, New Zealand, Pakistan	Effiom, Avoaja & Ohaeri (2012)
Scented perfume/lotions/detergents	Avoidance, Topical	USA, Canada, Australia, Brazil, Switzerland, Ireland, China, Sri Lanka	Douglas (2008)
Animal dung (cow, elephant)	Burning	N. America, Australia, India	Lale & Kulkarni (2010)
*Artemesia vulgaris* (L.) (Asian mugwort)	Burning, Planting	USA	Liu et al. (2013), Tripathi et al. (2000)
Ayurvedic leaves	Burning	Sri Lanka	McPartland (1997)
*Laurus nobilis* (L.) (bay leaves)	Burning	USA	
Coconut husks	Burning	USA, Australia	Kulkarni (2017)
Coconut shell with dhuna	Burning	India	Kulkarni (2017)
*Coffea Arabica* (L.) (coffee)	Burning	Europe, Brazil, USA, Africa	Satho et al. (2015)
*Larrea tridentate* (de Candolle) (Creosote)	Burning	USA	Green, Beroza & Hall (1960)
Dhup-an	Burning	Bangladesh	Sharma, Chauhan & Lal (2005)
Dried *Chrysanthemum spp.* plants	Burning	Kenya	Isman (2006)
Dry *Vitex negundo* (L.) (Nochi) leaves	Burning	India	Arutselvi et al. (2012)
Dry powdered rhizomes of certain plants	Burning	India	Choochote et al. (2005)
*Eucalyptus globulus* (Labillardiere) (Tasmanian bluegum) leaves	Burning	Brazil, USA	Maia & Moore (2011)
*Formes fomentarius* (L.) (tinder fungus)	Burning	Sweden	
*Poaceae* (L.) (grass)	Burning	Canada	Yoon et al. (2015)
Lemon scented candles	Burning	Netherlands	Jaenson, Garboui & Pålsson (2006)
*Prosopis juliflora* (DC.) (mesquite)	Burning	USA	Tibbets & Faeth (1999)
Mixture of diesel fuel & malathion	Burning	USA	Abdel-Sattar et al. (2010)
*Citrus sinensis* (L.) (orange) peel or dried rind	Burning	Europe, Africa, USA, Canada Brazil	Amusan, Idowu & Arowolo (2005)
*Citrus sinensis* (L.) (orange) peel with *Syzygium aromaticum* (L.) (clove)	Burning	USA	Zhu et al. (2001)
Peat	Burning	Sweden	Kettridge et al. (2014)
*Schinus terebinthifolius* (Raddi) (pepper tree)	Burning	Namibia	Karunamoorthi, Ramanujam & Rathinasamy (2008)
*Salvia officinalis* (L.) (sage)	Burning	USA	Ikeura, Kobayashi & Hayata (2012)
*Tamarix* (L.) (salt cedar)	Burning	USA	Hagler & Buchmann (1993)
*Santalum album* (L.) (sandalwood)	Burning	Australia	Amer & Mehlhorn (2006)
Spices such as *Cinnamomum* (Schaffer) (cinnamon), *Mentha* (L.) (mint), *Salvia officinalis* (L.) (sage), *Petroselinum crispum* (Miller) (parsley)	Burning	USA	Ishii, Matsuzawa & Vairappan (2010)
*Nicotiana tabacum* (L.) (tobacco) *or Cannabis sativa* (L.) (marijuana)	Burning	USA	Gozan et al. (2014)
Vanilla candles	Burning	USA	
Cigar smoke	Burning, Topical	USA, Ireland, Hungary	Jufri, Irmayant & Gozan (2016)
Kerosene	Burning, Unspecified	USA, Bangladesh, New Zealand	Pates et al. (2002)
Brewer’s yeast	Ingestion	Canada, New Zealand, USA	Bisseleua, Gbewonyo & Obeng-Ofori (2008)
Capsaicin	Ingestion	USA	Rogers (1984)
*Pelargonium citrosum* (Citronella) tea	Ingestion	Brazil, Mexico (herbal tea)	Maia & Moore (2011)
Dill pickles and *Allium sativum* (L.) (garlic)	Ingestion	Unknown	
Gin and tonic	Ingestion	Chile, New Zealand, South Africa	
*Zingiber officinale* (Roscoe) (ginger) tea	Ingestion	USA	Zhang, McAuslane & Schuster (2004)
*Citrus paradise* (Macfad.) (grapefruit) juice	Ingestion	USA	Yoon et al. (2015)
Iron pills	Ingestion	USA	
Local honey	Ingestion	USA	
Marmite	Ingestion	New Zealand	Bisseleua, Gbewonyo & Obeng-Ofori (2008)
*Citrus sinensis* (L.) (oranges)	Ingestion	Canada, USA	Amusan, Idowu & Arowolo (2005)
*Origanum vulgare* (L.) (oregano) oil	Ingestion	Canada	Licciardello et al. (2013)
Selenium supplements	Ingestion	USA	Angradi & Tzilkowski (1987)
Tonic water with quinine	Ingestion	USA, South Africa, Malaysia, France, New Zealand, Australia	
Alcohol	Ingestion, Avoidance	USA, Canada, Denmark, Hungary, Ireland	Lefèvre et al. (2010)
Apple cider vinegar	Ingestion, Spray, Topical	USA, Europe	
Aloe vera	Ingestion, Topical	South Africa, Italy, Germany, Norway, Australia	Subramaniam et al. (2012)
*Cinnamomum* (Schaeff.) (cinnamon)	Ingestion, Topical, Unspecified	USA, South Korea, Canada	Amer & Mehlhorn (2006)
Car Mobil	Larvicide	India	
Charcoal tabs in standing water	Larvicide	USA	Belant et al. (1997)
Clorox	Larvicide	USA	Aubernon et al. (2015)
Coffee disposal granules	Larvicide, Spray	USA, Brazil	Satho et al. (2015)
Diesel	Larvicide, Spray	Egypt, USA	Leverkus et al. (2017)
Cut a *Solanum lycopersicum* (L.) (tomato) in half and leave it next to the bed	Other	South Africa	Bleeker et al. (2009)
Cut *Syzygium aromaticum* (L.) (lemon)	Other	USA, Malaysia	Fradin & Day (2002)
Hanging dried *Eucalyptus globulus* (Labill.) (eucalyptus) branches	Other	USA	Pavela & Benelli (2016)
Hanging Ziploc bag of water with penny or lavender inside	Other	USA	
Jar of sugar water away from people	Other	USA	
Keep cut *Syzgium aromaticum* (L.) (lemon) in all the rooms	Other	Malaysia	Fradin & Day (2002)
*Tagetes minuta* (L.) (Khakibos) under mattress.	Other	South Africa	Brown, Ainslie & Beinart (2013)
Moth balls	Other	USA	Harris, Palmer & George (1983)
*Allium cepa* (L.) (onion) by side of the bed	Other	USA	Ueno et al. (2003)
*Pteridium aquilinum* (L.) (Bracken fern)	Plant	Canada	Donnelly, Robertson & Robinson (2002)
*Chrysathemum* (L.) (chrysathemums)	Plant	USA	Kamaraj et al. (2011)
*Eupatorium capillifolium* (Lamarck) (dog fennel)	Plant	USA	Tabanca et al. (2010)
*Equisetum* (L.) (horse tail)	Plant	USA	Bunescu, Florian & BODIŞ (2012)
*Monarda punctata* (L.) (horsemint)	Plant	USA	Tabanca et al. (2013)
*Lippia javanica* (Musudzungwane)	Plant	South Africa	Kruger et al. (2015)
*Achillea millefolium* (L.) (yarrow)	Plant	Canada	Moore & Debboun (2007)
*Tagetes* (L.) (Marigolds)	Plant, Larvicide	USA, Canada	Pavela & Benelli (2016)
*Solanum lycopersicum* (L.) (tomato plant)	Plant, Other	Austria, Switzerland, South Africa	Bleeker et al. (2009)
*Melissa officinalis* (L.) (lemon balm)	Plant, Topical	USA	Amer & Mehlhorn (2006)
*Rosmarinus officinalis* (L.) (rosemary)	Plant, Topical, Burning	USA, Australia, Hungary, Canada	Isman (2006)
*Nepeta cataria* (L.) (catnip)	Plant, Topical, Other	USA	Alem & Douglas (2004)
*Lavandula* (L.) (lavender)	Plant, Topical, Spray, Unspecified	USA, Germany, Canada, Australia	Amer & Mehlhorn (2006)
*Ocimum basilica* (L.) (basil)	Plant, Topical, Unspecified	USA, Australia, Ireland	Del Fabbro & Nazzi (2008)
*Pelargonium* (L.) (geraniums)	Plants	USA, Switzerland, Canada, Eritrea	Tabanca et al. (2013)
*Callicarpa americana* (L.) (Beauty berry)	Plants, Topical, Unspecified	USA, UK	Cantrell et al. (2005)
Beer, mouthwash, Epsom salt solution	Spray	USA	Lefèvre et al. (2010)
Boric acid (1%) and 10% sucrose solution	Spray	USA	Gore & Schal (2004)
Coconut oil and Dettol disinfectant	Spray	New Zealand	Sritabutra & Soonwera (2013)
Pinol (Mexican version of pinsol)	Spray	USA	Aubernon et al. (2015)
*Thymus vulgaris* (L.) (thyme) leaf tea mixed with *Pelargonium citrosum* (L.) (citronella) oil	Spray	USA	Isman (2006)
Water soaked *Fragaria ananassa* (Duchene) (strawberry)	Spray	Brazil	Ceuppens et al. (2015)
Listerine/mouth wash	Spray, Topical, Unspecified	USA, South Africa, Canada	Alexander et al. (1962)
Avon Skin So Soft	Topical	USA, Europe, Australia, South Africa	Rodriguez et al. (2015a)
Baby oil	Topical	USA	Akbar et al. (2005)
Baby wipes	Topical	USA	Akbar et al. (2005)
Bounce dryer sheet	Topical	USA, Saudi Arabia	
Cigarette butts soaked in alcohol	Topical	USA	Mondal et al. (2015)
*Syzygium aromaticum* (L.) (clove) in alcohol solution	Topical	Brazil	Plarre et al. (1997)
*Cocos nucifera* (L.) (coconut) oil	Topical	USA, Australia, Denmark, UK	Das et al. (2003)
Cream with *Melaleuca alternifolia* (Maiden & Betche) (tea tree) oil, *Eucalyptus globulus* (Labill.) (eucalyptus) oil, and bees wax	Topical	USA	Yang & Ma (2005)
Crushed dried *Carica papaya* (L.) (papaya) leaves	Topical	USA	Rawani et al. (2012)
Crushed *Hyptis suaveolens* (L.) (pignut)	Topical	Australia	Yang & Ma (2005)
Crushed *Mentha* (L.) (mint)	Topical	USA, Australia	Karamaouna et al. (2013)
Crushed seeds of *Lepidium sativum* (L.) (chandrashura), *Brassica nigra* (L.) (black mustard), and *Ricinus communis* (L.) (castorbean)	Topical	USA	Barone & Frank (1999)
Deer tallow	Topical	Alaska	Fargione & Richmond (1991)
Deodorant	Topical	New Zealand, USA	Verhulst et al. (2016)
Diatomaceous earth	Topical	USA	Islam et al. (2010)
Diluted fabric softener	Topical	USA	
Fresh aromatic leaves of *Myrtaceae* (Juss.) plants	Topical	Australia	Yaghoobi-Ershadi et al. (2006)
Germix	Topical	USA	
Lemon/lime juice	Topical	USA, Pakistan, Denmark	Amer & Mehlhorn (2006)
Local bear bread (a tree fungus, pollypore)	Topical	Alaska	
Mixture of baby oil and *Citronella* oil	Topical	Australia	Maia & Moore (2011)
Mixture of fresh or dried *Petroselinum crispum* (Mill.) (parsley) and apple cider vinegar	Topical	Unknown	
Mixture of garlic juice and crushed peels of limes and lemons	Topical	USA	
Mixture of vodka and citronella, geranium, and other essential oils	Topical	USA	Sakulku et al. (2009)
Mud	Topical	USA, Canada, Australia	
*Ananas comosus* (L.)(pineapple plant)	Topical	Canada	
Shampoo (as repellent)	Topical	Mexico, Malaysia, Britain, USA	
South African camphor oil ointment, called Zambuk	Topical	South Africa	Schearer (1984)
SPF 30 sunscreen	Topical	USA	Schueller & Romanowski (2016)
Talcum powder	Topical	USA	Mehr, Rutledgei & Morales (1985)
*Tanacetum vulgare* (L.) (tansey) leaves	Topical	Canada	Schearer (1984)
*Melaleuca alternifolia* (Maiden & Betche) (tea tree) oil	Topical	Ireland, USA, Norway, France, Australia	Ahmad, Aslam & Mamat (2016)
Thiamine patch	Topical	USA	Dua, Pandey & Dash (2010)
Tiger balm	Topical	USA, Cambodia, Australia, Malaysia	Sarwar et al. (2017)
Vanilla extract	Topical	USA	
Vaseline	Topical	South Africa, USA	Lindqvist, Lindqvist & Tiilikkala (2008)
*Azadirachta indica* (Juss.) (neem)	Topical, Burning, Plant	India, USA, Bangladesh, Eritrea, Brazil, West Indies, Germany, Nigeria	Isman (2006)
Vicks Vaporub/menthol	Topical, Other	USA, Canada, Australia, South Africa, Namibia	Alankar (2009)
*Mentha piperita* (L.) (peppermint) lotion/oil	Topical, Other (camping tent)	USA, Unknown	Geetha & Roy (2014)
Mixture of baby oil, menthol, and Dettol	Topical, Spray, Unspecified	Australia, New Zealand	Bunker & Hirschfelder (1925)
Crushed *Backhousia citriodora* (F.Muell.) (lemon myrtle) or *Eucalyptus globulus* (Labill.) (eucalyptus) leaves	Topical, Spray, Unspecified	Australia	Maia & Moore (2011)
Vinegar/vinegar based solutions	Topical, Spray, Unspecified	USA	Rahmat et al. (2014)
Crushed up leaves of the *Myrica cerifera* (L.) (Wax Myrtle tree)	Topical, Unspecified	USA, Australia	Cilek, Hallmon & Johnson (2010)
*Aleurites moluccanus* (L.) (Kukui nut) oil	Topical, Unspecified	USA (Hawaii)	Nakayama & Osbrink (2010)
Rubbing alcohol	Topical, Unspecified	USA, South Korea	Govere et al. (2000)
Witch hazel	Topical, unspecified	USA, Canada	
Baking soda	Unspecified	USA	Beresford et al. (1996)
*Citrus bergamia* (Risso.) (bergamot) oil	Unspecified	USA	Peterson & Coats (2001)
*Actaea racemose* (L.) (black cohosh)	Unspecified	USA	
*Plantago major* (L.) (broadleaf plaintain)	Unspecified	Germany	
Broken liquid aspirin	Unspecified	USA	Alem & Douglas (2004)
*Melaleuca cajuputi* (Thomas Powell) (cajuput) oil	Unspecified	Indonesia	Amer & Mehlhorn (2006)
*Calendula officinalis* (L.) (English marigold) oil	Unspecified	Canada	Tavassoli et al. (2011)
*Capsicum annuum* (L.) (cayenne)	Unspecified	USA	Wimalaratne et al. (1996)
Chlorine	Unspecified	USA	Mathis & Quarterman (1953)
Copper pins	Unspecified	Germany	Becker, Oo & Schork (2015)
Diluted Raw Armor’s starch	Unspecified	USA	
Hairspray	Unspecified	USA	
*Jasminum* (L.) (jasmine)	Unspecified	Ireland, Spain	Amer & Mehlhorn (2006)
Key lime	Unspecified	USA	
Mixture of *Cymbopogon* (Kurt Sprengel) (lemongrass) oil, mouthwash, and *Prunus dulcis* (Mill.) (almond) oil	Unspecified	USA	Ansari et al. (2000)
Mixture of Listerine and witch hazel	Unspecified	USA	Ansari et al. (2000)
Mixture of *Melaleuca alternifolia* (Maiden & Betche) (tea tree) oil, apple cider vinegar, and water	Unspecified	USA	Di Campli et al. (2012)
Mixture of WD-40, camphor/phenol, and mineral oil	Unspecified	New Zealand, USA	
Nail polish	Unspecified	USA	Govere et al. (2000)
Oil paint	Unspecified	Pakistan	Kareru et al. (2010)
*Pogostemon cablin* (Blanco) (patchouli) oil	Unspecified	USA, Canada	Maia & Moore (2011)
*Pinus sylvestris* (L.) (pine) oil	Unspecified	USA	Maia & Moore (2011)
Pine tar	Unspecified	USA	Thorsell et al. (1998)
*Sesamum indicum* (L.) (sesame) oil	Unspecified	USA	Trongtokit et al. (2005)
*Mentha spicata* (L.) (spearmint) oil	Unspecified	USA	Ansari et al. (2000)
Sulphur	Unspecified	USA, Canada	Ferraro (1995)
Thieves oil	Unspecified	USA	Dua, Pandey & Dash (2010)
Toothpaste	Unspecified	USA	Geetha & Roy (2014)
Trader Joe’s Tea Tree Tingle	Unspecified	USA	Ahmad, Aslam & Mamat (2016)
Windex	Unspecified	USA	Miller (1983)

### Unconventional mosquito repellent methods

The major goal of this study was to collect information on unconventional mosquito repellent and control methods. In one of the last survey questions, participants were asked to list additional repellent methods that were not previously mentioned. Table 1 shows 167 of the unconventional methods that survey participants listed. The table is organized into the type of survey responses, followed by the application/preparation of the product mentioned, and the regions from where the participants that listed it reside at the time of the survey. The last column shows scientific references for this particular method. If the product mentioned was used in multiple ways, the most commonly listed application was listed first. In general, we documented the use of various plants, smokes, personal hygiene products, household chemicals, softeners, diets, supplements, and other behaviors. Within this collection of mosquito repellent strategies, the most commonly listed method was topical application of various substrates including personal hygiene products, plant rubs, and household chemicals. The second most listed unconventional strategy was the use of smoke produced by incineration of various materials encompassing specific local plants, tobacco products, and candles. Following the burning strategy was ingestion of various food items and supplements. Using live plants as a spatial repellent was the fourth most commonly listed method we documented as unconventional. Many of these plants are known as sources for essential oils.

For some items we documented several ways of use. For example, garlic was used either by ingestion, applying to the skin, or creating a spray that was used as an outdoor spatial repellent. In some cases we documented conflicting attitudes toward certain interventions; for example both the ingestion, as well as the avoidance of ingestion of bananas as well as alcohol were listed as mosquito-avoidance strategies.

One of the most reoccurring responses was the use of dryer sheets. Over 200 participants have used dryer sheets of some sort as a repellent device. Not listed as frequently as dryer sheets but having participants from a diversity of regions mention it, were products of the Neem tree (*Azadirachta indica (Jussieu)*) and coffee. Neem products were used by either burning or topically and their use was stated by people from Brazil, USA, India, Nigeria, Eritrea, and Bangladesh. Coffee was listed as either being burned or used grounded on lawns or as a larvicide. Coffee was listed by participants that resided in Greece, South Africa, Brazil, United Kingdom, Canada, or the United States. Burning sage and planting and/or topical application of catnip were listed only by respondents currently residing in the US.

## Discussion

The data that we collected from this survey were acquired with the intent to find unconventional methods of mosquito control or repellency. We were able to identify 167 different methods.

### Unconventional mosquito control methods

Some of the unconventional methods reported include *Aloe vera* (L.) and grapefruit, both of which have been previously studied. *Aloe vera* (L.) in combination with bio-control agent *B. sphaericus* has shown strong larvicidal effects against *A. aegypti (L.) larvae* (Subramaniam et al., 2012). Grapefruit oil was tested as a repellent against the adult rice weevil, *Sitophilus oryzae (L.)* ( Yoon et al., 2007). Other methods warrant efficacy tests such as key lime, sugar consumption, and iron pills. Many of these unconventional methods can be further analyzed in order to identify the chemical component(s) that make them a repellent such as analyzing the chemical ingredients in specific toothpastes or Windex for their repellency effects.

Many of our respondents used DEET and citronella candles which is interesting because DEET is a very effective mosquito repellent while citronella candles are not (Rodriguez et al., 2017; Rodriguez et al., 2015a). These popular methods could be used as controls in future studies. Picaridin and other commercial insect repellent products were mentioned but not as often as DEET and citronella.

The participants listed many different methods which were used at varying degrees in different regions. This may be due to the regional availability of certain mosquito repellent products or regional preferences for a certain method. It was unexpected to find a high number of participants from North America listing bed nets as a method that they’ve used. Interestingly, many of the North American bed nets users noted that they used them abroad.

### Limitations of this study

Despite the fact that the survey was available in three different languages—English, Spanish, and Portuguese—the overwhelming numbers of answers were received from English-speaking survey respondents. A higher percentage—around 75%—of the survey respondents held some sort of academic degree (associates, bachelors, or graduate) (Fig. 1). Also, we received a total of 5,209 responses with 3,645 of those responses from people currently residing in the United States. Therefore, our respondent pool was strongly biased towards English-speaking academics residing in the US (see Supplemental Information 3). This suggests that we were unable to successfully plant “community-based seeds” both in the US and other countries in which the survey would be shared outside the academic community. The high number of female participants in this survey study could indicate that the topic of mosquito control appeals more to females than to males. An alternative explanation is that females are more likely to respond to online surveys in general which is supported by other studies like the one done by Sax, Gilmartin & Bryant (2003), where both paper and online surveys were more heavily answered by females than males.

### Challenges and future directions

Survey research presents some challenges for fully understanding how alternative repellents are used ( Krosnick et al., 2015). Our survey responses tended to include ingredients rather than practices. Some responses may have left out specific actions or ingredients that respondents did not consider important. Future research may further explore these uses through collaboration with anthropologists and other field researchers who can observe and document actual practices as well as interview key informants with particular expertise in these alternative practices, including traditional healers and community elders. The intent of this study was to identify potential affordable, accessible, and culturally relevant mosquito control practices that can be made widely available to the public. However, one ethical issue that will need to be addressed in the future research is the protection of traditional knowledge and the potential for exploitation of that knowledge for commercial gain (Mugabe, 1999).

Availability, choice, use, and effectiveness of repellents and other mosquito avoidance strategies are also important variables for mathematical models predicting mosquito-borne disease dynamics. The concept of ‘One Heath’ integrates human, animal and environmental health. This multidisciplinary approach is relevant for veterinarians, ecologists, biologists, and others to understand and predict the spread of disease among people, animals, and within the environment (Destoumieux-Garzón et al., 2018). One Health approaches will benefit from solid knowledge on local repellent use, and its efficacy. Thus, knowing how different people in different regions defend themselves against mosquitos, and knowing the efficacy of these methods, can be combined with other data to contribute to disease outbreak predictions (Benelli & Duggan, 2018).

In conclusion, we identified several understudied mosquito repellent methods that may or may not be more effective and practical across diverse global settings. In many cases the efficacy of these methods has not been evaluated in scientific experiments. This study offers opportunities for further research into unconventional mosquito repellent methods to determine their effectiveness. Those that prove to be effective may be taken up more quickly by populations at risk for contracting mosquito-borne diseases because local populations may have better access to them and they may fit better into the context of people’s daily lives.

##  Supplemental Information

10.7717/peerj.5151/supp-1 Supplemental Information 1 Survey questions

10.7717/peerj.5151/supp-2 Supplemental Information 2 Raw data This file contains the individual answers of the survey participants.

10.7717/peerj.5151/supp-3 Supplemental Information 3 Geographic distribution of all survey responses This file shows the number of responses from all the countries from which there were responses.

## Additional Information and Declarations

### Data Availability

The following information was supplied regarding data availability: The raw data are provided in the Supplemental Files.
