# Supplementary material for: An online survey of personal mosquito-repellent strategies
Source: PeerJ. 2018 Jul 3;6:e5151. doi: 10.7717/peerj.5151 (PMC6034598; doi:10.7717/peerj.5151)
Supplement: Supplemental Information 1 [file peerj-06-5151-s001.docx]

**Question 1:** What is your sex/gender? (*Multiple choice*)

- Female
- Male
- Other

**Question 2:** What is your age? (*Multiple choice*)

- 18-37
- 38-57
- 58 or older

**Question 3:** What is the highest level of education you have completed? (*Multiple choice*)

- Less than high school
- High school only (no college)
- Some college (no degree)
- Associate’s degree (or equivalent)
- Bachelor’s degree (or equivalent)
- Graduate degree

**Question 4:** Please list your current state, country and/or region of residence and up to three additional places you have lived the longest. (*Open-ended*)

**Question 5**: Which of the following best fits how you MOST OFTEN think about mosquitos? (*Multiple choice*)

- I rarely think about mosquitos.
- I am just a little concerned about getting mosquito bites.
- I am just a little concerned about getting a disease from a mosquito.
- I am very concerned about getting mosquito bites.
- I am very concerned about getting a disease from a mosquito.

**Question 6**: Which of the following best fits your knowledge of mosquitos? (*Multiple choice*)

- I have conducted research on mosquitos.
- I regularly read scientific research about mosquitos, but I haven’t conducted research on mosquitos.
- I regularly read popular media and/or news articles about mosquitos, but not scientific research.
- I have learned about mosquitos from family and friends, but do not regularly read about mosquitos.
- I know about mosquitos only from my personal experience with them.
- I know very little about mosquitos.

**Question 7:** In this study, we are interested in learning about non-commercial methods of repelling or controlling mosquitos. Please list all methods you have ever used to control or repel mosquitos. (*Open-ended*)

**Question 8:** Please review the list below and check any methods that you have used that you did not already list above. [Check all that apply]

- Vitamin B patches
- Mosquito repellent patches
- Mosquito Dunks (or other BT product)
- Medications (such as vitamin B or garlic pills)
- Insecticide-treated clothing
- Sonic repellers
- None of the above
- Mosquito repelling plants
- Insecticide spray (such as permethrin)
- Mosquito bracelets
- Essential oils
- Bed nets
- Mosquito coils
- Electric insect zapper
- Spray-on natural repellents
- Citronella candles
- Spray-on mosquito repellent with DEET

**Question 9:** Please rank up to the top five most effective methods from the all methods you have ever used. (*Open-ended*)
